# SOCIAL SUPPORT, MENTAL HEALTH, AND SEXUAL ORIENTATION: A STUDY OF THREE TIME POINTS IN THE CLSA

**DOI:** 10.1093/geroni/igad104.2573

**Published:** 2023-12-21

**Authors:** Alexandra Grady, Arne Stinchcombe

**Affiliations:** University of Ottawa, Ottawa, Ontario, Canada; University of Ottawa, Ottawa, Ontario, Canada

## Abstract

Lesbian, gay, and bisexual (LGB) older adults exhibit mental health disparities stemming from a lifetime accumulation of minority stress experiences. Psychological resources like social support may buffer against these poor mental health outcomes. The purpose of this study was to examine indicators of depression and social support by sexual orientation over time in a sample of Canadian adults ages 45-85. We used data collected at three time points in the Canadian Longitudinal Study on Aging (CLSA; baseline, 2011-2015; follow-up 1, 2015-2018; and follow-up 2, 2018-2021). Depression was measured using the Center for Epidemiologic Studies Depression scale (CESD-10) and social support was measured using the Medical Outcomes Study Social Support Survey (MOS-SSS). For depression symptoms (n=38,244; 830 LGB), results indicated a significant effect of sexual orientation (F(2, 38242)=35.93, p<.001) such that LGB participants had more depression symptoms than heterosexual participants. There was also a significant effect of time on depression symptoms (F(2, 38242)=26.07, p<.001); mean depression scores decreased over time. For social support (n=35,795; 770 LGB), results indicated a significant effect of sexual orientation (F(2, 35794)=45.31, p<.001) such that LGB participants reported lower levels of social support than heterosexual participants. There was also a significant effect of time (F(2, 35794)=5.98, p=.003); the mean scores for social support increased over time. The potential role of social support in moderating depression symptoms will be discussed. This study adds to our understanding of potential protective factors influencing the mental health of older LGB people.

